# Supplementary figures and images for: ATP Purinergic Receptor P2X1-Dependent Suicidal NETosis Induced by Cryptosporidium parvum under Physioxia Conditions
Source: Biology (Basel). 2022 Mar 14;11(3):442. doi: 10.3390/biology11030442 (PMC8945010; doi:10.3390/biology11030442)

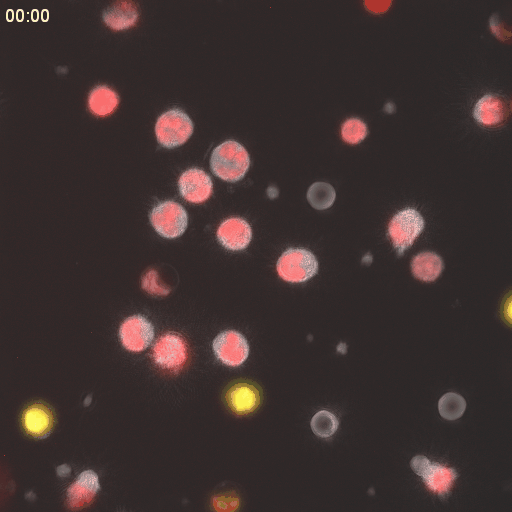

Supplement: Supplementary file 1 [file biology-11-00442-s001.zip › Supplementary files/suplementary video 3.gif]
